# How to do Better N400 Studies: Reproducibility, Consistency and Adherence to Research Standards in the Existing Literature

**DOI:** 10.1007/s11065-021-09513-4

**Published:** 2021-08-09

**Authors:** Anđela Šoškić, Vojislav Jovanović, Suzy J. Styles, Emily S. Kappenman, Vanja Ković

**Affiliations:** 1 Teacher Education Faculty, University of Belgrade, Belgrade, Serbia; 2 Laboratory for Neurocognition and Applied Cognition, Department of Psychology, Faculty of Philosophy, University of Belgrade, Belgrade, Serbia; 3 Division of Psychology, School of Social Sciences, Nanyang Technological University, Singapore, Singapore; 4 Centre for Research and Development On Learning (CRADLE), Nanyang Technological University, Singapore, Singapore; 5 Singapore Institute for Clinical Sciences (SICS), A*Star Research Entities, Singapore, Singapore; 6 Department of Psychology, San Diego State University, San Diego, CA USA

**Keywords:** ERP methodology, N400, Pictures, Event related potentials, Open science, Reproducibility

## Abstract

Given the complexity of ERP recording and processing pipeline, the resulting variability of methodological options, and the potential for these decisions to influence study outcomes, it is important to understand how ERP studies are conducted in practice and to what extent researchers are transparent about their data collection and analysis procedures. The review gives an overview of methodology reporting in a sample of 132 ERP papers, published between January 1980 – June 2018 in journals included in two large databases: Web of Science and PubMed. Because ERP methodology partly depends on the study design, we focused on a well-established component (the N400) in the most commonly assessed population (healthy neurotypical adults), in one of its most common modalities (visual images). The review provides insights into 73 properties of study design, data pre-processing, measurement, statistics, visualization of results, and references to supplemental information across studies within the same subfield. For each of the examined methodological decisions, the degree of consistency, clarity of reporting and deviations from the guidelines for best practice were examined. Overall, the results show that each study had a unique approach to ERP data recording, processing and analysis, and that at least some details were missing from all papers. In the review, we highlight the most common reporting omissions and deviations from established recommendations, as well as areas in which there was the least consistency. Additionally, we provide guidance for a priori selection of the N400 measurement window and electrode locations based on the results of previous studies.

**Supplementary Information:**

The online version contains supplementary material available at 10.1007/s11065-021-09513-4.

## Introduction

*Event-related potentials*, or ERPs, are fluctuations in voltage that are associated in time with a physical or mental trigger (e.g., an external stimulus, a thought), and which can be recorded from the human scalp using electroencephalography (Picton et al., 2000). According to Luck (2014), ERPs were most likely first recorded in 1939 by Pauline and Hallowell Davis, who were investigating differences in the activity of the brain during wakefulness and sleep (Davis et al., 1939; Davis, 1939). Since those early days, ERP analysis has become a method of choice to answer a variety of questions about normal and pathological functioning of the human brain. The number of papers accumulated over the past decades is huge – for example, just a search for the exact phrase “event related potential*” on the Web of Science gave 26,047 results (on November 05, 2019), in fields ranging from psychiatry, immunology or even obstetrics, to psycholinguistics and educational psychology.

The rise in popularity of the method and its availability to laboratories across the world has increased the need for clear practice guidelines and standards that are widely available. The first guidelines for ERP recording were published in 1977, derived from the International Symposium on Cerebral Evoked Potentials in Man held in Brussels in 1974 (Donchin et al., 1977), updated by the Society for Psychophysiological Research in 2000 (Picton et al., 2000), and again in 2014 in a broader report focusing on electroencephalography as well as magnetoencephalography (Keil et al., 2014). In addition, specialized guidelines have been developed for fields that require a distinct approach, such as clinical studies (Duncan et al., 2009; Kappenman & Luck, 2016) or experiments with children (Taylor & Baldeweg, 2002), and ERP methodology papers have been published to provide guidelines for answering specific questions (e.g. Boudewyn et al., 2018; Delorme et al., 2007; Junghöfer et al., 1999; Kappenman & Luck, 2010; Tanner et al., 2015). Methodology books on the ERP technique have also been published to help new researchers get acquainted with the basics and provide a more thorough overview (Handy, 2005; Luck, 2005, 2014).

These publications have provided useful guidance to researchers on how to make methodological decisions they encounter in ERP experiments. However, while basic standards outline what is *not acceptable*, there are still many decisions to make when recording and analysing ERP data, and for each of them, multiple options are acceptable. This necessarily puts a researcher in a dilemma over which way to go and opens a possibility of intentional or unintentional data manipulation in order to fit results to expectations.

An example of this issue is described in a recent paper by Luck and Gaspelin (2017), who demonstrated how “researcher degrees of freedom” could influence statistical analysis of ERP data. ERP recordings typically employ dozens of electrodes and result in hundreds of time points, which results in an almost unlimited variety of possible data analysis approaches, and, consequently, in the probability of a false significant finding approaching certainty.

These issues are not just a theoretical concern, as it has been demonstrated recently when a large collaborative preregistered replication attempt (Nieuwland et al., 2018) failed to support the key findings of an influential, widely cited study on the N400 in response to articles and nouns (DeLong et al., 2005). The study by Nieuwland et al. and ensuing commentaries (DeLong et al., 2017; Yan et al., 2017) do not only highlight the importance of careful design of new studies and replication attempts, but they also provide further evidence of the sensitivity of ERP analysis to subtle methodological decisions. Namely, Nieuwland et al. (2018) report that one of the issues raised after publishing a preprint of their paper was the difference in baseline duration between the original study by DeLong et al. (2005) and their replication attempt. The discrepancy in methods section resulted from omission of baseline information from the paper by DeLong et al., and it was corrected after communication between the two author teams following preprint publication. This example demonstrates the importance of ERP data analysis choices and comprehensive reporting on these choices.

The problem of researcher degrees of freedom is not unique to ERP methods – on the contrary, it has been recognized in other fields as well (Gelman & Loken, 2013), and it is particularly concerning in studies involving abundance of data that can be treated in a multitude of ways (for a general discussion of the problems associated with researcher degrees of freedom, see Chambers, 2017). Neuroscience studies are especially prone to the problems of researcher degrees of freedom, due to the information-dense nature of the data collected, and the myriad of possible pre-processing pathways. For instance, one review of methods reporting in fMRI (Carp, 2012) has shown that there are almost as many analyses pipelines for fMRI data as there are individual studies, and many papers fail to provide sufficient information on methods to allow precise independent replications.

### Present Study

Given this variability of methodological options, and the potential for them to influence study outcomes, it is important to understand how published ERP studies have been conducted in practice and to what extent researchers are transparent about their data collection and analysis procedures.

The aim of our paper is, thus, to provide a comprehensive overview of the present state of the field, as a platform from which to develop guidance for future neurocognitive research. The questions of interest are (1) how much methodological variability exists among studies investigating a well-established neurophysiological phenomenon, which would be expected to follow almost the same procedure, (2) which practices are the most prevalent, (3) how often researchers deviate from guidelines for good practice, (4) which deviations are the most common, (5) how often descriptions of methods and analyses are insufficiently detailed, and (6) which are the principal areas where improvements in reporting practices are necessary. Answering these questions allows us to provide evidence-based guidelines for making decisions about the analysis pipeline, for example, when a priori decisions are made based on previous research (e.g., choosing a reference site or the measurement time window). This overview also provides the opportunity to caution researchers against the most common deviations from best practices in ERP methodology and reporting.

Papers included in this review span over three decades, and many things have changed in the way ERP data is collected, processed, and analysed since then – new technologies and analyses have become available and we have learned new things both about ERP methodology and the N400 itself. This is reflected in changes between different versions of guidelines for good practice (Donchin et al., 1977; Keil et al., 2014; Picton et al., 2000). Therefore, the review also includes an insight into trends over time, to investigate how improvements in ERP methodology and recommendations were reflected in practice.

ERP study methods, pre-processing and analysis pathway depend to some extent on the study design, for example, on which components are being measured, the modality of the stimuli, and the population from which subjects are recruited. Given this variability, we chose to focus on a narrow category of ERP studies, those investigating a well-established component (the N400) in the most commonly assessed population (healthy neurotypical adults), in one of its most common modalities (visual images). The N400 is a negative-going wave peaking at about 400 ms, whose amplitude is larger after presentation of a stimulus whose probability of occurrence is low within its semantic context (Kutas & Federmeier, 2011). For example “He spread the warm bread with socks” would elicit a larger N400 than “He spread the warm bread with butter” (Kutas & Hillyard, 1980). It is a well-known ERP component with a long history of successful conceptual replications (Kutas & Federmeier, 2011), making it an ideal target for investigations of methodological and analytical coherence in the field. Thus, the findings of the review are directly relevant to a large group of N400 researchers, and some points also may generalize to other ERP components.

In order to provide the most robust dataset from which to draw conclusions, we conducted this survey of the existing literature in the form of a systematic review. The review provides an extensive insight into a variety of parameters, including properties of the study design (e.g., sample size), data pre-processing (e.g., filtering procedures), measurement (e.g., N400 time window), statistics (e.g., electrode sites in the ANOVA model), and, for more recent papers, references to supplemental information (e.g., raw data or analysis codes).

### Objective

This systematic review documents the diversity of methodologies used, and clarity of reporting in peer-reviewed ERP papers, reporting an N400 to a visual image and recorded in adult healthy participants, published between January 1980 – June 2018 in journals included in two large databases: Web of Science and PubMed.

## Method

Protocol of this study was not registered online, but we followed the Preferred Reporting Items for Systematic Reviews and Meta-Analyses (PRISMA) guidelines (Moher et al., 2009), where it was applicable. The PRISMA checklist for our review is available in Supplement 4 of the OSF repository for this article (Šoškić et al., 2021; see Supplementary materials for more information).

### Database Search

The first step was to search online databases for papers relevant for this review. Two large aggregated databases were chosen: Web of Science and PubMed. These two databases contain a large sample of ERP studies, which is likely representative for the majority of peer-reviewed ERP literature.

Each database was searched using the following search terms: (*N400* or *ERP N4*) AND (*visual stimuli*, *visually evoked potentials, drawing(s), image(s), photo(graph-ies,y,s)* or *picture(s)*). Default settings for search engines were used on both platforms including the search for key words in all fields and automatically generated MeSH (Medical Subject Headings) terms for PubMed, and search within Topic for the Web of Science. A list of exact search phrases with numbers of hits for each conducted search is available in the OSF repository for this article (Supplement 1). Search was limited to papers published after 1980, the year of the N400 discovery (Kutas & Hillyard, 1980). It took place on 11th July 2018, and included papers published until 30th June 2018.

All references were merged into a single database using Mendeley Desktop (Mendeley Ltd.) to identify duplicate publications from the two sources.

### Article Scanning

Following the PRISMA procedure, in order to identify which of the unique articles returned by the search did indeed contain an N400 study relevant for our review, we screened each article for possible inclusion. Two researchers independently conducted the screening, and where ambiguity or disagreement between the independent screeners arose, additional team members were asked to clarify or expand the initial criteria for eliminating studies.

The main criterion for selection was that the papers were original research papers on studies that included an ERP experiment with images as stimuli, and where the N400 following image onset was examined. Studies which included simultaneous presentation of information in various modalities or rapid presentation of visual image stimuli were not considered due to an effect of such designs on the N400 properties and analysis. For the same reason, papers were excluded if they involved any interventions or recording equipment which could affect experimental methodology or data analysis (e.g., tDCS, fMRI). Studies were selected for analysis only if participants were adults with no reported history of psychopathology.

On the other hand, we imposed no limitations regarding methods or treatment of outcome measures, since we focused on methodology, and not on results. We also included 15 studies that involved tasks with other types of target stimuli in addition to the task with visual images. Finally, there was no upper limit for participant age. The N400 is known to change linearly with age (Kutas & Iragui, 1998), so any cut-off point would have been arbitrary. Furthermore, it was relatively common for studies in our sample to have at least one or two middle-aged participants. As a result of this decision, we included two aging studies with elderly participants.^1^

The review was limited to articles in English, since the majority of papers on ERPs are published in this language. However, studies conducted in other languages, but reported in English, were also included in the pool. Additionally, the focus of this review was on papers that had been verified and accepted by the scientific community via formal peer review. For this reason, we did not look for papers that were not published or at least in press at the time of the search. Furthermore, we checked all included papers for retractions and corrections. Conference proceedings were included in the pool if they were full-sized papers, whereas short resumes or abstracts were excluded due to the typical lack of methodological detail in the short format.

In addition, references were excluded if they could not be located through their journal or web search. Publications were considered duplications and duplicates were excluded if multiple papers had the same study design, sample characteristics, and same statistical results. In cases where papers, potentially or expressly, reported different analyses of the same data, all versions were included. Since we focused on methods, these papers added new information to our review, and they overlapped only in study design and pre-processing, which would likely have been the same if the authors had collected new data for each analysis.

### Data Extraction

All papers were independently assessed by two researchers, who reported the results in separate spreadsheets. The two spreadsheets were then merged, and all diverging or unresolved points were jointly analysed by one of the authors working on papers assessment and a third team member. When a conclusion about a reported item could not be reached due to conflicting, insufficient or ambiguous information, it was labelled as “inconclusive”. In the case of some variables, categories could not be made in advance. In these cases, descriptions were logged and merged using the procedure above, and categorization was carried out post hoc by one team member.

Data was extracted for the following properties, using a total of 74 columns (variables):

- *experimental design:* design description, smallest sample size^2^ – total and per group, smallest number of trials – total and per situation, jittering pre-stimulus intervals, use of techniques to prevent overlap between the overt response and ERP window;
- *equipment:* hardware used for EEG recording (cap, amplifiers, other), software used during the experiment and data pre-processing and analysis (stimulus presentation, EEG acquisition, EEG/ERP pre-processing, statistics, other);
- *data recording and pre-processing:* reference used in data analyses, recording montage (active sites), scalp electrodes impedance, basic low-pass and high-pass online and offline filter settings (cut-off, roll-off, and cut-off type – half-amplitude or half-power), use of notch filters, number of trials left after trial rejection – what type of information was reported and what were the values, baseline length, epoch duration and whether it overlapped with an overt response or the beginning of the next trial, which artifacts were eliminated, artifact identification and elimination procedures, whether the order of operations could be assumed based on the description;
- *measurement:* N400 time window, and the reason for selecting this specific window, amplitude measure;
- *statistical analyses and data presentation*: which electrodes or electrode constellations were analysed (analysis montage), electrode analysis strategy (basis for choosing analysis montage), main analysis approach (e.g., ANOVA model), additional analyses (e.g., post hoc tests, topographical analyses), whether there was correction for sphericity violation and having multiple statistical tests, number of uncorrected (M)AN(C)OVAs, how many other components were analysed in addition to N400, which additional components were analysed and whether they were earlier or later than the N400, whether negative was plotted up or down in the graphs;
- *about publications:* publishing year, authors, whether it was a conference proceeding or a journal article;
- *general*: a column for additional data and comments.

Finally, *availability of supplemental data* (e.g., stimuli, raw data), identifiable through the article, was examined. This is a more recent trend in scientific reporting, and we did not expect most papers to provide this information. However, there has been a push in the past few years towards improving reproducibility and credibility of research through encouraging open science practices (Ioannidis et al., 2014; Nosek et al., 2015), so we were interested whether more recent papers had started to implement these recommendations.

Due to the volume of information, variable descriptions and coding details are provided in a separate file in Supplementary materials (Codebook—Supplement 3).

### Data Analysis

The results were summarized by examining descriptive statistics. Frequencies of categorical variables, as well as means and standard deviations of numerical variables. In rare cases, where it was not possible or rational to categorize papers due to extreme variability, verbal descriptions were summarized by examining frequencies of key words.

Conveniently, 25 papers included in this review (18.9%) were published between 1988–2000, when the first detailed guidelines for ERP research were published (Picton et al., 2000), and the same number of publications came out since 2015, a year after presenting the latest version of the guidelines (Keil et al., 2014). We present a brief comparison of these two groups, to show how improvements in ERP methodology and recommendations were reflected in practice.

## Results and Discussion

### Database Search and Article Selection

In total, 1508 papers were returned by the searches. Two additional references were added, which were found during a preliminary stage of the systematic review, but they did not show up in database search results. After merging search results and removing duplicates, 790 titles remained.

Of these, 625 articles were excluded on inspection of title and abstract, and 33 were excluded after inspecting the full text. Alltogether, 17 of the papers which were excluded were in languages other than English,; three references were excluded because they could not be located through their journal or web search,; one paper was eliminated because it was a duplicate publication, 83 papers did not include an ERP N400 experiment (e.g., theory papers, intracranial recordings), others were rejected based on their methods (sample or study design). As a result, 132 papers survived the exclusion criteria.

There were no retractions, and only one correction (concerning a name spelling error). Six conference proceedings were included in our review, and the remaining articles were peer-reviewed journal articles.

**Fig. 1:**
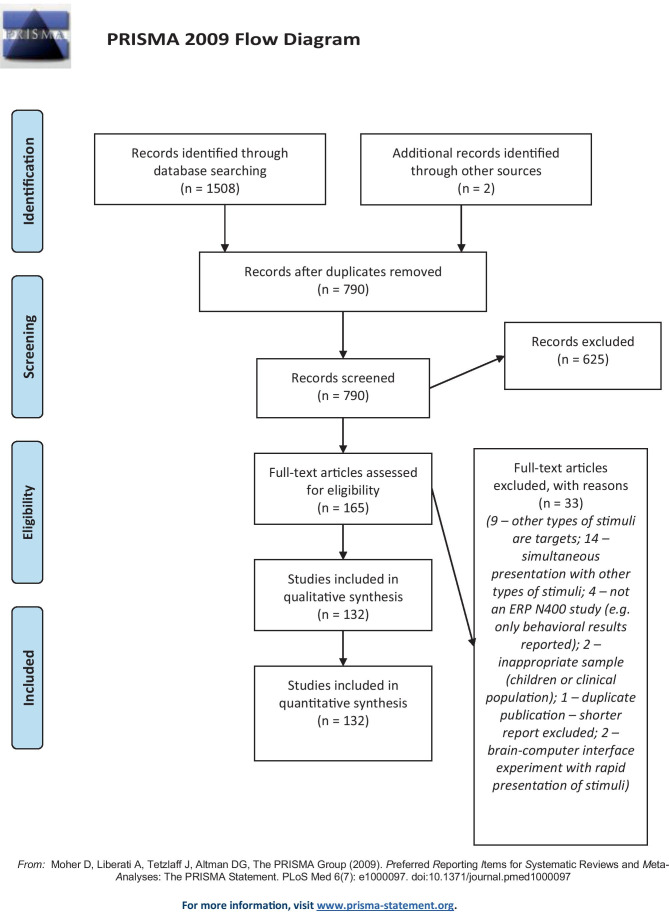
PRISMA flow diagram

The PRISMA flow diagram summarizing articles included or excluded at the different stages of screening can be seen in Fig. 1. Supplement 2a contains libraries with references found by searching PubMed and Web of Science. The full list of all papers included in this report can be found in Table 1, and a library with all references selected for analysis is available in Supplement 2b. Supplement 5 contains the spreadsheet with extracted information on individual papers, while Supplement 6a, b, c, d contains files with all analyses and graphs presented here.

### The Big Picture

#### How Often are Descriptions of Methods and Analyses Insufficiently Detailed? Which are the Principal Areas Where Improvements in Reporting Practices are Necessary?

It would not be difficult to guess which were the most frequently described aspects of the reviewed studies. *Sample size,*^3^* number of presented trials,* and *amplitude measurement window*, *types of statistical analyses* (e.g., ANOVA) were reported universally or almost universally, with only a few exceptions.

Similarly, *amplitude measure* was reported in 93.2% of papers, and the *analysis montage* could be extracted from 88.6% of all papers. These numbers are high, but still concerning, given that these are some of the most important aspects of a study.

At the next level of clarity, there were methodology decisions which were described in the majority of papers, but there was still a considerable number of papers in which this information was either missing or not adequately described. First, information about the *reference* used for data analysis was provided in 80.3% of all papers. The most frequent issue with reporting on the voltage reference was not providing a description of the recording montage when using the average reference, although, in some cases, details about a mastoid or earlobe reference were omitted, too. While omitting details about the mastoid reference can be relatively benign, the average reference can differ a lot depending on the recording montage (Luck, 2005, 2014), and it may even be inappropriate to use it depending on the recording montage size and electrode locations (Junghöfer et al., 1999; Keil et al., 2014; Picton et al., 2000). Additionally, in some papers, it was difficult to assess whether the term “linked reference” referred to physical linking or averaging. Similarly, *baseline* duration was explicitly described in 77.3% of papers. Some of the papers which did not contain baseline duration information, included reports on pre-stimulus period duration, but the two may not necessarily be the same, and they were not the same in other papers included in this review. Additionally, we did not quantify frequencies of issues related to graphical representation of ERPs, but it is noteworthy that in some papers, baseline period was not shown in graphs, either in its entire duration or at all. Epoch durations were provided slightly more often, in 83.3% of all cases. It was similar with reporting impedances for low input-impedance amplifiers (84.0%), but descriptions of data quality obtained by high-input impedance amplifiers were provided only in four out of ten papers (42.9%). Amplifier manufacturer and recording montage were both provided in 59.8% of cases. The latter was in some cases completely left out from the reports, but other papers were labelled inconclusive because of conflicting information, usually between figure, electrode list and electrode count. Recording montages often have dozens of electrodes, which can make errors easy to overlook, so future researchers may want to make sure to double-check whether all information is correct and consistent. Almost a third of all papers (28.0%) did not describe the methods for eliminating artifacts beyond specifying whether they were removed using correction or rejection. Even when more details were given, they were not always sufficient to evaluate and replicate the procedure. Important decisions about data analysis – selection of time window(s) and electrode locations for the main statistical analysis – were not justified in about a third of all cases (34.0% and 36.3%, respectively). Moreover, when previous literature was cited as the sole basis for these decisions, in about half of all cases (47.8%), they were not supported by the cited papers. In addition, various details about the analyses applied to these time windows and electrodes were inconclusive in 4–17% of papers. In some of these cases, some information was omitted, but, in others, there was conflicting information between Methods and Results sections. One possible cause of this discrepancy could be the peer review process. Therefore, future researchers may want to check whether the appropriate changes were made in all parts of the text if a different approach is taken after feedback from reviewers.

Finally, there were aspects of the examined studies which were rarely adequately described, and which warrant urgent attention of researchers and reviewers. When it comes to the *number of trials per condition which were averaged together,* 13.64% papers reported the average number or percentage of rejections for each condition, along with the range of trial counts or at least the threshold for excluding a participant, while 40.2% publications had no information on the number of trials which was left after rejection due to artifacts and/or behavioural errors. Reports on digital and especially analog *filters* frequently specified only their cut-off frequencies (54.1–96.2% of cases for different filters), and even the cut-off was described without specifying whether it represents half-amplitude or half-power point in the frequency response function in 78.8% papers. A reconstruction of the *order of pre-processing and measurement steps* could be made in 46.2% of all cases, and in many of these cases, it was only an assumption based on the order in which the operations were described. Three common issues can be noted (1) in some papers, the new reference after re-referencing was specified in the recording section together with online reference, (2) a pre-processing step that had likely taken place (e.g. artifact removal) was not mentioned in the paper, so a reader could not be sure if it had taken place and at which stage, (3) the last step, averaging, was described first, in a sentence in which several other steps were mentioned as side points, in a way that made it impossible to tell at which moment they were applied. Finally, we did not quantify this, but it was not possible to determine *how many comparisons* were made in total in some of the studies.

To summarize all variables, 61 papers (46.21%) were categorized as inconclusive or contained details labeled as inconclusive on variables containing verbal descriptions. In addition, at least some details were omitted from all papers. However, even when filter properties other than cut-off, equipment, and software (the most commonly omitted items) were not taken into account, there were only two studies in which all other information was provided (conducted by Cansino et al. (2012) and by Federmeier & Kutas (2002)). 

**Fig. 2:**
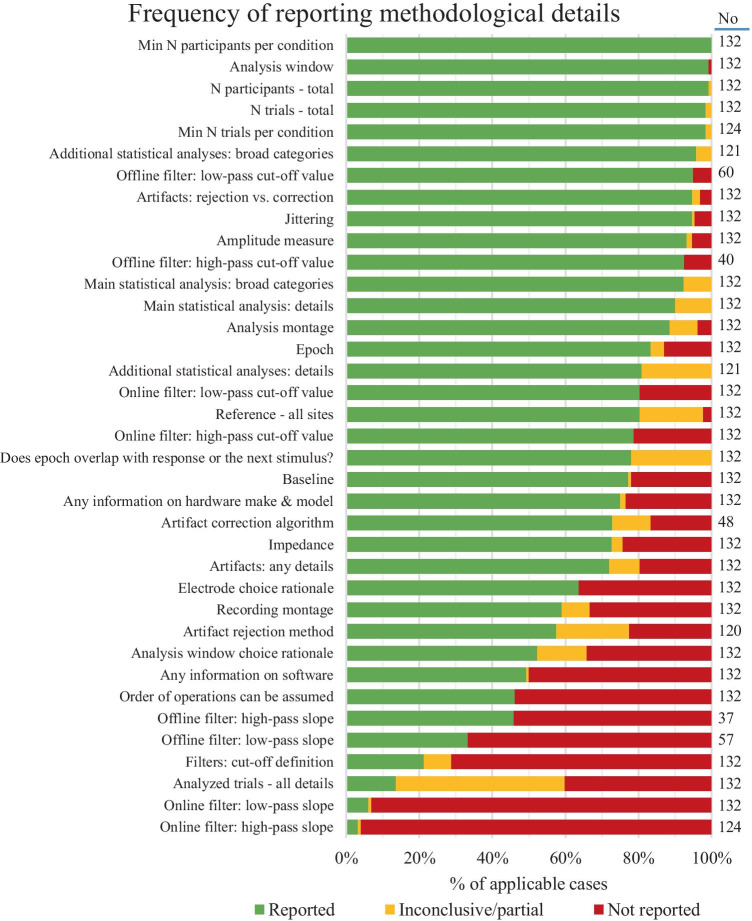
Frequencies of omitting methodological details from reports. The *y* axis shows methodological information that was examined, while the *x* axis shows the percentage of papers in which this information was provided, partly provided, or not provided. All percentages are relative to No – the number of cases relevant for the variable in question (e.g., studies in which a procedure was used). Green bars show percentage of papers in which the methodological information in question was provided. Yellow bars show percentages of papers in which some information was given, but it was either partial or inconclusive. Red bars show percentages of papers from which the detail in question was omitted. Table of frequencies and more details on them can be found in Supplement 6d

This information is graphically summarized in Fig. 2. The figure shows percentages of papers in which (1) the methodological information in question was provided, (2) some information was given, but it was either partial or inconclusive, or (3) the detail in question was omitted. For more details about Fig. 2, see Supplement 6d.

Aside from the report itself, little supplementary material was identifiable through analysed papers, even for more recent studies. Most papers (86.4%) did not refer to accessible supplementary materials other than reports on additional analyses. Admittedly, in one of them, readers were informed that data was stored on a departmental server and could be accessed by contacting authors or the department, while another paper provided a link to a Harvard Dataverse page, albeit locked to website visitors even after registration. Additionally, 9.8% papers provided only lists of stimuli descriptions, and another 2.3% provided actual stimuli or information needed to identify them in published databases of images. There were, in fact, only two papers in which access to ERP data had been provided – a link to behavioural and raw ERP data in one paper, and to component mean amplitudes in the other. There were no studies with published codes for stimulus presenting, ERP data pre-processing or analyses. To provide supplementary information has become both possible and advocated (through Open Access initiatives) only recently, so high availability in the entire sample of papers cannot be expected. The question of supplement availability in the more recent studies is covered in the section Trends over time.

#### How Much Variability is There Among Studies that Would be Expected to Follow Similar Procedures, Because They All Investigate the Same Well-Established Neurophysiological Phenomenon? Which Practices are the Most Prevalent?

**Fig. 3:**
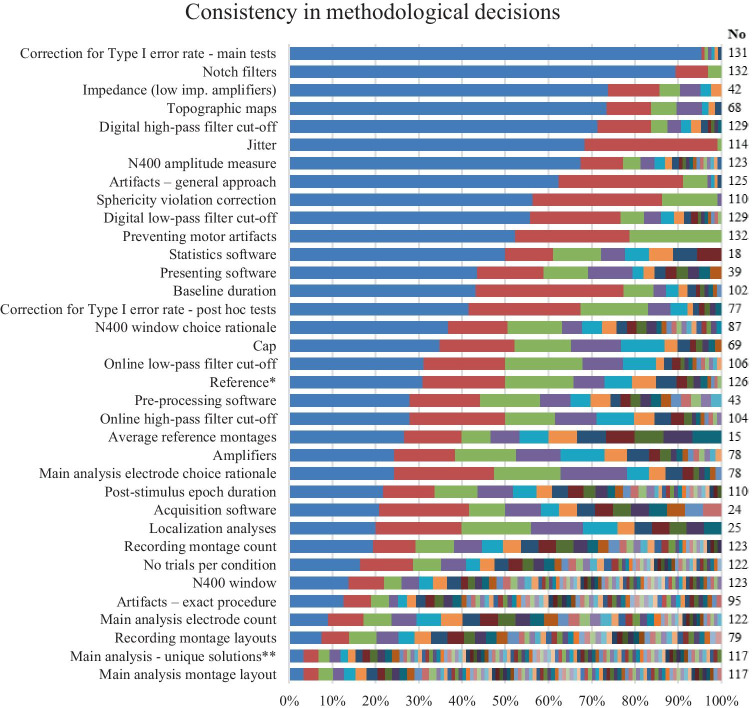
Variability and frequencies of different choices when making methodological decisions. The *y* axis shows methodological information that was examined, while the *x* axis shows the percentage of each option. All percentages are relative to No – the number of cases relevant for the variable in question (e.g., studies in which it was possible or appropriate to apply a procedure, or in which the relevant information was available). More details can be found in Supplement 6d. *Note:* * average reference is grouped into one option in this row; ** unique combinations of electrode layouts and their groupings into factors

While Fig. 2 presented how many papers reported on different methodological decisions, Fig. 3 shows frequency of each option for a given methodological decision, when this information was available.

There were several points on which the majority of researchers took the same approach. The decision which was present in the largest majority of papers was that main effects and interactions were treated as a priori* comparisons*, and thus not subjected to correction for multiple comparisons (more than 95% of all papers), despite the number of comparisons which were made in most studies. Next, in approximately nine out of ten papers, ANOVA was the *statistical analysis* of choice. *Notch filters* were avoided in nine out of ten papers, as well. When low-impedance amplifiers were used, authors reported lowering *impedances* below 5kΩ in 73.8% studies and even lower in additional 4.7% papers (78.5% in total). Out of 64 papers which provided *maps of topographic distribution* of ERPs, 78.1% opted for the most common option – voltage maps. Mean *amplitude* (calculated from single and difference waves) and variations of the mean mastoid/earlobe *reference* were used in three quarters of papers. The latter is especially relevant to future researchers who want to present their data in a way comparable to the previously conducted studies. If average or other less frequent references are used, the future researchers may want to include at least plots based on mean mastoid/earlobe reference, too, to enhance comparability with previous research (Picton et al., 2000). In a total of 70.0% of papers, authors reported testing for *sphericity* and applying corrections where necessary, and 80.5% of them used the more conservative Greenhouse–Geisser adjustment (1959). When it comes to trial design, seven out of ten studies did not rely on stimulus timing *jittering* (71.1%) or measures to *prevent overlap between motor response and ERP components* (52.3%), to reduce sources of noise in ERP recordings. Examples of measures used to prevent overlap between motor response and ERP components included a cue for participants to respond only after the ERP time window had passed (which is efficient only if combined with jittering the cue because of preparatory motor activity) and designs in which there was no overt response to stimuli used in the N400 analyses, either because overt responding was not required or because the participants responded to other stimuli. When it comes to *artifact elimination method*, 62.4% papers reported rejecting all types of artifacts which were detected. Analyses based on LORETA were most frequently used to *estimate sources of ERP components*, although three distinct types of LORETA analysis were found (LORETA, sLORETA, swLORETA, used in 16.0–20.0% localisation analyses).

The next group of methodological decisions were the ones on which the reviewed publications diverged, but the number of options was moderate and at least some common options could be identified. Such decisions were *equipment manufacturer* (12 and 18 manufacturers with 34.8% and 24.4% share for the main option for cap and amplifiers, respectively), *software* used in different stages from stimulus presentation to statistical analysis (between 8–17 options, and 20.8–50.0% share for the main option), *baseline* (11 different baselines, but 100 ms was used in 43.1% of all cases), *high-pass and low-pass filter cut-offs* (9–18 different cut-offs, but 0.1 and 30 Hz were the most frequent; note that digital high-pass and low-pass filters were used in 28.7% and 44.2% of publications, respectively), *time window selection strategy* (11 strategies, out of which visual inspection was the most common, and it was the sole or deciding factor in 50.6% of cases in which the window selection strategy was reported, and a third of all papers), *method of selecting electrodes for the main statistical analysis* (11 options, the two most commonly reported strategies were analysing all recorded channels without grouping, 24.3%, and visual inspection, 23.1% of cases in which the strategy could be identified), and post hoc* comparisons* (no correction in 42.9% of all papers in which post hoc tests were described, and 9 different corrections, out of which Bonferroni and Tukey HSD were the most frequent). A borderline case in this category of variables was *epoch duration,* which included 32 different epochs, but the 1000 ms one was used in 21.8% of all cases.

Finally, there were methodological decisions to which almost every team of authors took a different approach. When it comes to *specific methods of artifact detection and elimination*, 67 unique pipelines were found, each of them used in only one paper or a handful of publications. Regardless, as long as artifacts are properly eliminated from the trials used for averaging, all these artifact detection strategies, despite their variability, should produce comparable outcomes. Another decision on which publications diverged was the *recording electrode montage*: 50 different layouts of between 1 – 144 electrodes (34 different montage sizes) were identified in papers in which this information was provided. The most frequently used montage was found in six papers. The average montage had 46.33 electrodes (SD = 36.08), while the most common montage size was 64 electrodes (19.5% of papers in which this information was available). In some cases, electrode montages are fixed, but in the future, part of the variability in recording site montages could be reduced by considering consistency with previous literature when selecting electrode locations for recording when this is relevant (e.g., average reference, full scalp analyses). When it comes to the average reference, it was not possible to determine how many different electrode montages were used to produce it, because the montages were not described in half of these papers. Still, it can be seen based on the reported montage sizes, that there were at least 14 different montages in 27 papers in which the electrode montage was reported, with as little as 19 or as many as 144 electrodes. Therefore, the topographic distributions of effects obtained from these montages, especially those with fewer than 64 electrodes, differ to an unknown extent (see Junghöfer et al., 1999; Keil et al., 2014; Luck, 2005, 2014; Picton et al., 2000). The number of trials per condition also varied widely between studies. As few as 6 and as many as 400 trials were presented per condition in the reviewed studies (M = 60.78, SD = 51.57, 49 different options), and about half of all studies (56.6%) had between 20–50 trials per condition. As a result of predominantly data-dependent strategies for the analysis window selection, the N400 amplitude was measured from 69 different latency ranges, 76.8% of which were used in a single study. Similarly, the N400 effect was determined based on 66 different electrodes combined into 93 unique sets, of 41 different sizes varying between 1 – 144. Furthermore, these sets were subjected to 99 different main statistical analyses.

What could a future researcher rely on to make an a priori decision about statistical comparisons, given this variability in the N400 measurement window and measurement electrodes choice? To answer this question, we extracted latencies and electrode locations from individual experiments to extract the overlapping time points and electrode locations.

**Fig. 4:**
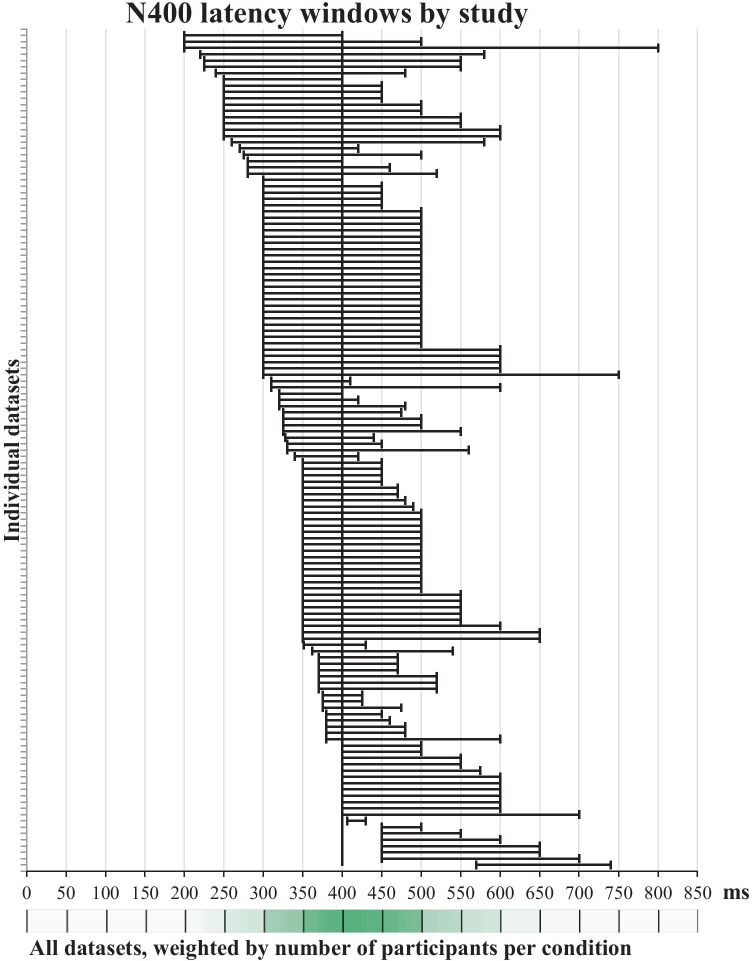
N400 window choices in all datasets, i.e., experiments on separate participant groups, in papers in which an N400 analysis window was reported (N = 133 datasets from 120 papers from which both sample size and latency window could be extracted). If a paper reported multiple analysis windows or multiple experiments on the same subjects, it was represented by a single window, whose lower and upper bounds were the most extreme measures of all windows reported in this paper. Bands show N400 latency ranges for all individual datasets. The heat bar in the bottom displays frequency of including each time point (1 ms) in the N400 latency range, weighted by the number of participants per condition for each dataset. Shades of green show differences between the lowest (white) and the maximum weighted frequency (dark green). This graph has been created by modifying the template made available by Neyeloff et al. (2012)

Regarding measurement window choices, latency ranges for a total of 133 experiments were extracted from 120 papers which had information on both sample size and N400 latency range. Next, each millisecond in the 0–850 ms post-stimulus epoch received a score based on the number of times it fell within the N400 range and the number of participants per group in the experiments in which it was found. The results showed that there was a sudden drop in scores after 500 ms, and that there were two large increases – after 300 and 350 ms. The increase following 300-ms point was slightly larger compared to 350 ms, and 300–500 ms was also the most frequently used measurement window. Therefore, if future researchers wanted to select their N400 measurement window a priori based on the existing literature, the 300–500 ms window would be the option most supported by previous literature, at least in the case of experiments with pictures as target stimuli. Figure 4 shows all latency ranges that were used for the N400 measurement and analysis in the reviewed literature, and its heat bar is a visual representation of the weighted frequencies for all time points. Supplement 7d contains a more detailed description of this analysis, while Supplement 6c contains an Excel version of Fig. 4 with all scores for the heat bar.

In order to investigate the variability in analysis montage choices, we examined which electrodes were reported in studies in which up to 12 electrode sites were analysed. As explained in the Codebook (Supplement 3), this cut-off point was chosen because montages with more than 12 electrode sites typically involved analysing all or most of the recorded sites, which were distributed over the entire scalp, while the smaller recording and analysis montages were more frequently targeted on the N400 effect location.

**Fig. 5:**
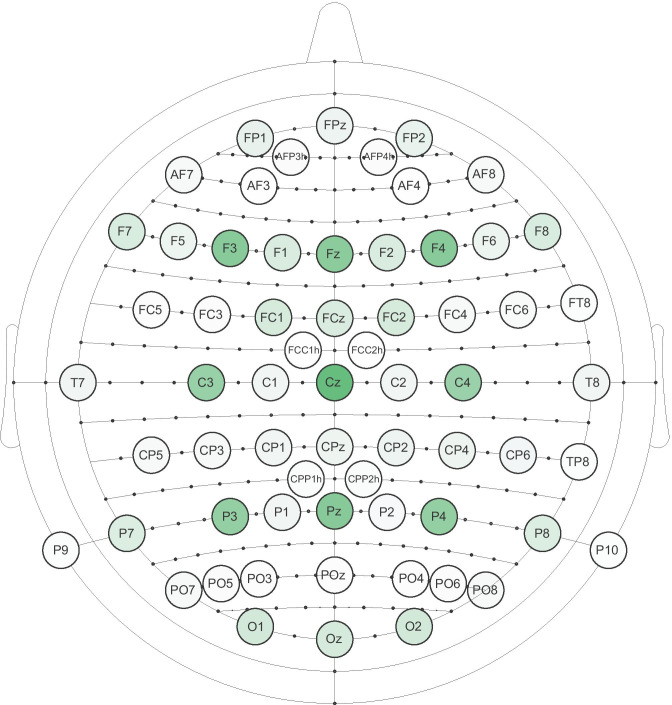
The montage shows all electrodes that were used for measurement of the N400 in the main statistical analysis, regardless of the reference point. Only studies with 12 or fewer electrodes were used to generate this montage, because larger montages more frequently included analyses of the entire scalp with broadly distributed electrodes. If a paper included more than one experiment with different subjects, both experiments were included in the analysis separately. Shades of green show differences between the lowest (white) and the maximum frequency (dark green) of using an electrode, weighted by the number of participants per condition for each experiment

For this purpose, data on 65 experiments conducted on different samples was extracted from 58 publications. Within analysis montages used in these experiments, 66 different channels were found. Frequency of using each channel for analysing data from the selected 65 experiments was registered, and, additionally, this information was weighted by the number of participants per group. All electrodes used in the analyses are shown in Fig. 5, in which weighted frequency of each site is presented using colour scale. More information on this analysis can be found in Supplement 6d, while the Excel calculations can be found in Supplement 6a. Nine electrodes stood out compared to others: F3, Fz, F4, C3, Cz, C4, P3, Pz, and P4. Each of these electrodes was used in 23 or more experiments, compared to all other sites, which were included in analyses of 10 or fewer experiments. The results were the same when data was weighted by the number of participants per group. Notably, no electrode appeared in more than about a half of all studies: Cz was the electrode most commonly used for the N400 measurement, compared to the other eight sites, and it was included in 55.4% cases.

The described variability can be partly attributed to differences in the recording montage, but not entirely, given that montages frequently overlapped on many electrode sites. The variability was more likely the consequence of the method of electrode location selection, which was frequently data-driven and often allowed for researcher degrees of freedom (for more information on channel selection strategies, see Supplement 7d).

One shortcoming of the previous analysis is the variability in references used to measure the N400 effect, as its topographic distribution varies depending on the reference. As it was shown earlier, most of the references were mastoid, a smaller proportion average, and other references were infrequent, so Fig. 5 is most heavily influenced by these two references. Therefore, the montage shown in Fig. 5 shows the variability of the electrode location choices in this field, but it would not be the best grounds for making a priori decisions in future studies. To provide guidance for deciding on the analysis montage based on previous literature, we repeated the same analysis of electrode locations, but only for 24 experiments reported in 18 publications which reported using a mastoid or earlobe reference, which are expected to yield the same distribution. Only cases in which it could be verified that the reference was not physically linked were included, because physical linking would also influence topographic distribution. In total, 47 different electrodes were found, and the most frequent choices were F3, Fz, F4, C3, Cz, and C4. They were used in 12–14 experiments and stood out the most when frequencies were weighted by the number of participants per group. Like in the case of the analysis of all experiments, P3, Pz and P4 were also frequent, but they did not stand out this time. Each was used in 8 experiments, while other electrodes were used in 1–6 experiments, and the weighted frequencies were closer to the rest of electrodes than to F3, Fz, F4, C3, Cz, or C4. To summarize, future researchers who use mastoid, earlobe or similar references, and want to select electrodes for the N400 measurement a priori based on the previous literature, should pick F3, Fz, F4, C3, Cz, and C4. Like in the case of the previous analysis, Excel sheets with all frequencies and calculations can be found in Supplement 6a.

Due to the variability in montages used to create the average reference and the number of papers using other references, it was not possible to provide specific guidelines for montages other than mastoid/earlobe.

#### How Often Do Researchers Deviate From Guidelines for Good Practice? Which Deviations are the Most Common?

While “it depends” how many participants and trials are needed for a sufficiently powered study, as Boudewyn et al. (2018) put it, it is safe to say that studies with fewer than ten *participants per condition* and studies in which no more than thirty *trials per condition* were averaged together were underpowered to detect smaller within-group and between-group effects. There were 11.4% studies in the first group, and 28.6% of studies in the second group.

Among the **recording and pre-processing steps**, a few issues were found. Inappropriately high high-pass *filters* (≥ 0.3 Hz half-amplitude or half-power, see Luck, 2014; Tanner et al., 2015), either analog or digital, were found in 10.6% of cases, while inappropriately low low-pass filters (< 20 Hz half-amplitude, see Luck, 2014) were found in 7.6% of publications (for a discussion about half-amplitude vs. half-power cut-off, see Supplement 7c). Linked mastoid or earlobe *references* were used in about a quarter of all studies (23.0%, assuming that the description of recording with a linked reference was correct;(for issues with using linked references, see Keil et al., 2014; Miller et al., 1991; Picton et al., 2000). When average reference was used, the montages were not always sufficiently large and distributed over a large area of the head, as it is recommended (Junghöfer et al., 1999; Keil et al., 2014; Picton et al., 2000). While all *baseline* durations were appropriately long (100 + ms), some studies may benefit from extending the baseline from 100 to 200 ms. This could enhance amplitude measurement stability, especially if the N400 latency range extends beyond 500 ms (Luck, 2014). Other baseline-related issues included showing waveforms before baseline correction in graphs and noise or confounding activity in the baseline period. It is difficult to assess prevalence of deviating from the best practices in *artifact detection and correction* due to limited information available and diversity of methods which were described, but suboptimal strategies were found in some cases (e.g., rejecting trials exclusively based on a fixed base-to-peak threshold, for a discussion about artifact elimination methods, see Luck, 2014). Finally, not all reported *orders of pre-processing steps* were optimal, or in some cases even acceptable (e.g., if high-pass filtering was applied after averaging).

The aspect of the reviewed studies which warrants the most attention is data analysis, more specifically, *Type I error rate probability*. There were several decision points which contributed to the high probability of finding a false positive result in the reviewed studies. As mentioned earlier, strategies for selecting the time window and electrodes for the N400 measurement were frequently data-dependent, despite the relatively stable latency and spatial distribution of the N400 (see Kutas & Federmeier, 2011). Data-dependent strategies are not an issue per se, as long as appropriate corrections for multiple comparisons are being made (e.g., mass univariate approach; see Groppe et al., 2011). However, the papers included in this review frequently opted for strategies such as visually inspecting waveforms to select time windows and electrodes, combined with subjecting the same waveforms to statistical analyses appropriate only for a priori comparisons. Such strategies are sometimes called “double-dipping” because they involve relying on the same dataset to select a subset of data to be analysed and also conduct the analysis (Kriegeskorte et al., 2009). As Luck and Gaspelin (2017) explain, such approaches include implicit and practically uncorrected comparisons that are being made prior to analysing data, and it is not appropriate to apply statistical analyses such as ANOVA on subsets of data selected this way as if the selection had been made a priori, because the Type I error rate is compromised. The second major point that contributed to the Type I error rate inflation was the number of analyses which was conducted. For example, out of 115 papers which used ANOVA, ANCOVA or MANOVA for the N400 analysis, 70.4% papers had more than one (M)AN(C)OVA model without correction for multiple comparisons, and more than a half (53.9%) had more than four such models (M = 7.12 models, SD = 10.35). The total number of uncorrected models went up to 576 (one for each experimental factor, electrode site and short window) in one study. Additionally, the N400 was not the only component that was analysed in 88.4% of publications – between 1–14 additional components were analysed in these studies (M = 2.63, SD = 2.58). When the number of components is multiplied by the number of analyses employed to investigate them, as well as with the number of factors in each analysis, the number of comparisons becomes so large that it is not appropriate to treat main effects as a priori comparisons. Taking all this together, it is urgent that ERP field makes a shift towards more appropriate data analysis strategies in the future.

Finally, some practices are not deviations from guidelines for good practice but adopting alternatives more broadly may benefit future studies. Three such practices were registered: *jittering* inter-stimulus interval, *delaying motor response* with a cue to respond to avoid overlap with ERP components if combined with jittering, and boosting statistical power by *lowering impedances* even when high-input impedance amplifiers are used (for more information about impedances, see Kappenman & Luck, 2010).

#### Trends Over Time

As shown in Table 1, the oldest paper included in this review was published in 1988. Reflecting growth in ERP use, the papers are not distributed evenly over the years. Instead, their number grew over time. Approximately a half of all papers (50.8%) were published in the last ten years, since 2010.

In this section, we will present a brief comparison between the 25 papers published between 1988–2000, when the first detailed guidelines for ERP research were published (Picton et al., 2000), and the 25 publications which came out since 2015, a year after presenting the latest version of the guidelines (Keil et al., 2014), to show how improvements in ERP methodology and recommendations were reflected in practice.

##### Study Design and Sampling

Several aspects of study design have changed over time. First, the more recent studies had more participants per condition (M_old_ = 15.36, n_new_ = 18.52), even though between-group designs, which are less powerful, were more frequent in the older literature (f_old_ = 24%, f_new_ = 8%). The contemporary studies also had more trials per condition, even after excluding two studies, one in each group, which had unusually large numbers of trials per condition (M_old_ = 39.38, M_new_ = 50.74, excluding outliers). The two groups of studies did not differ a lot, however, when it comes to reporting on how many trials were averaged together – about half of papers in both groups did not report outcomes of artifact rejection, although the number was slightly higher in the sample of older papers (f_old_ = 56%, f_new_ = 44% for not reporting). Jittering interstimulus or intertrial interval became more widespread over time (f_old_ = 12%, f_new_ = 40%), while self-paced timing was more frequent in the older literature (f_old_ = 16%, f_new_ = 0%). Authors of the earlier studies used both tasks with delayed response and no response to the N400-eliciting stimulus as a method to eliminate brain activity related to motor response equally (f_no response_ = 20%, f_delayed response_ = 20%, f_neither_ = 60%), while delayed motor response was a preferred solution in the more recent studies (f_no response_ = 8%, f_delayed response_ = 32%, f_neither_ = 60%).

##### Apparatus and Software

Equipment and software were more frequently described in the more recent publications (cap reports: f_old_ = 28%, f_new_ = 76%; amplifiers reports: f_old_ = 44%, f_new_ = 76%; software reports: f_old_ = 0–20%, f_new_ = 36–68%, depending on the category). In addition to more recent guidelines recommending more detailed reports, the increase in software reporting can likely be attributed to more recent development of widely available commercial and open-access software packages, as well as more complex procedures for data processing and analysis, offered by these packages.

##### Recording and Pre-Processing

The older publications reported impedances more frequently than more recent ones (f_old_ = 80%, f_new_ = 64%). This is related to the fact that high-impedance amplifiers were often used in the contemporary studies (f_new_ = 40%), but none of the authors of the more dated papers reported using such equipment. As explained in the section on impedances, papers on studies in which high-impedance amplifiers were used, did not contain alternative data quality indicators when impedance information was not available.

Recording montages have become bigger since the early studies. The average number of electrodes in the montage increased form M_old_ = 13.38 to M_new_ = 55.04. Montage sizes in the older papers were also more diverse, while 4 out of 10 the more recent studies were recorded with 62–64 active channels.

Voltage reference of choice has also changed over time. Linked mastoid or earlobe references were often used in the early studies (f_old_ = 56%), while other solutions were diverse and infrequent. In the latest studies, linked references have been abandoned for superior offline references, mean mastoids (f_new_ = 40%) and average reference (f_new_ = 28%). In case of the latter, the authors described the recording montage in only one paper.

Expansion of digital filtering tools allowed filtering data with a narrower bandpass offline. Among the older publications, five had reports on low-pass digital filters and one mentioned high-pass filtering. In contrast, data was filtered digitally in more than half of the more recent studies (f_high-pass_ = 56%, f_low-pass_ = 64%). Online filters were described in all of the older publications. The more recent papers, however, usually only had descriptions of analog filters when digital filters were not used. Only 3 out of 16 contemporary papers which mention digital filters also included information on analog filters. Roll-off was described by 8% older and 24% of the more recent papers, and it was provided for offline filters in all cases but one. Cut-off type was specified for all filters in 60% of the older publications, and in 12% of the more recent ones. Even though almost all sources (Cook & Miller, 1992; Keil et al., 2014; Luck, 2005, 2014; Picton et al., 2000) advise against notch filters, they have not been abandoned yet (f_old_ = 12%, f_new_ = 16%).

Similarly, development of better artifact correction algorithms and increased availability of programs which implement them resulted in a shift from primarily rejection (f_old_ = 88%) to combining rejection with correction (f_old_ = 32% for rejection, f_new_ = 48% for combined methods).

Baseline duration differed between the old and the new papers, too. Data was most frequently baseline-corrected relative to 200 ms baseline in the new studies (f_100_ = 24%, f_200_ = 52%), and relative to 100 ms in the oldest studies (f_100_ = 44%, f_200_ = 20%).

Unfortunately, descriptions of the order of operations have not become more precise (in f_new_ = f_old_ = 64% of papers, the order of operations could be at least assumed).

##### Measurement and Analysis

While reporting on the measurement analysis window has changed, the main strategy to choose it has not. The contemporary papers included rationale for choosing analysis window more frequently (f_old_ = 48%, f_new_ = 64% for reports that did have it) and used multiple different arguments to justify the choice more often (f_old_ = 0%, f_new_ = 20%). The main strategy in both groups was visual inspection (f_old_ = f_new_ = 32%). Although this is understandable in the case of early papers, when there were not many options for data analysis or previous studies to provide grounds for specific hypotheses, the most recent guidelines advocate against this practice (Keil et al., 2014). Mean amplitude was the main amplitude measure in both studies (f_old_ = 68%, f_new_ = 64%), while the use of peak amplitude has decreased (f_old_ = 28%, f_new_ = 12%).

Conversely, frequency of reporting on selection of electrodes for the main statistical analysis has not changed (old: f_not reported_ = 48%, f_inconclusive_ = 4%; new: f_not reported_ = 40%, f_inconclusive_ = 4%), but the most frequently used analysis strategy has. The most common approach in early studies was to avoid selecting electrodes for analysis by treating all recorded channels as levels of one factor (f_old_ = 28%, f_new_ = 12%), while the contemporary studies rely on visual inspection more often (f_old_ = 4%, f_new_ = 28%). Like recording montages, analysis montages have also increased (M_old_ = 11.37, M_new_ = 21.76). Consequently, the risk of Type I error has increased with time. This risk was reduced on a different front: more recent papers had fewer (M)AN(C)OVA models (M_old_ = 10.14, M_new_ = 4.22^4^; papers with only one model: f_old_ = 8%, f_new_ = 32%), as well as fewer ERP components taken from the same waveforms (M_old_ = 2.76, M_new_ = 2.12; papers with only one component: f_old_ = 44%, f_new_ = 80%).

Regarding visualization of spatial distribution, maps have become more widespread (f_old_ = 2%, f_new_ = 44%). Topographic distribution analyses have also changed. In the group of older papers, PCA analysis was used in two studies (8%), and it has not been used in the more recent ones. On the other hand, there were four more recent publications (16%) in which LORETA-based analyses were employed.

##### Overall Reproducibility

Overall, the two groups of studies had similar frequencies of omitting methodological details or presenting them in an ambiguous way. The average contemporary study had some inconclusive information on 1.6 out of 70^5^ variables, and some information was omitted in 14.92 out of 70 cases on average. Similarly, the older publications had 1.52 variable values with inconclusive and 16 values with missing information.

Providing supplementary methodology materials has become more frequent, although not a norm, in line with the Open Access movement and wider options for storing research data online. Sharing at least brief descriptions of stimuli has become more frequent (f_old_ = 8%, f_new_ = 16%). On top of this, two of the most recent studies (8%) have also published some of their ERP data, albeit only mean component amplitudes in one case.

### The Detailed Picture

Given the number of variables and papers covered in this study, a thorough report on all results surpasses the format of a journal article. In the Big Picture section of this paper, we have attempted to provide an overview of the main findings, but readers interested in a more detailed account of available guidelines and our results regarding any aspect of ERP methodology included in this study, can find them in supplementary materials linked below:

1. **Study design and sampling** (Supplement 7a): experiments and factors; trial structure and timing; sample size; number of trials (presented and included in analyses).
2. **Equipment and software** (Supplement 7b).
3. **Recording and pre-processing** (Supplement 7c): impedance; recording montage (active sites); reference and re-referencing; filtering (high-pass and low-pass filters cut-off and roll-off, other filters); baseline; poststimulus epoch (length and overlap with overt response or the next stimulus); eliminating artifacts; order of operations.
4. **N400 amplitude measurement and statistical analysis** (Supplement 7d): amplitude measurement (grounds for choosing analysis window, latency range, amplitude measure); main statistical analysis of the N400 amplitude (grounds for choosing electrode locations; which sites were chosen for the main analysis; analysis); additional analyses of the N400 component; correction for Type I error rate and other corrections; topographic distribution analyses and visualization; general considerations regarding measurement and analysis.

## Conclusion

What should be the main takeaway from this study? While this review has highlighted some of the shortcomings of the existing N400 literature, our goal was not to show that all studies have issues. It is likely that there are no perfect studies, as ERP data recording, processing and analysis are incredibly complex processes, and our analysis of trends over time has shown that many aspects of ERP methodology and reporting have improved over time. Moreover, these very standards we have today, which were cited in this study, result from continuous endeavours by the ERP research community to improve methods and analyses of ERP data. Many concerns which were discussed here are not unique to ERP research – on the contrary, they are shared with similar fields of study, such as fMRI, psychophysiological recordings, and, in some respects, even behavioural research. This study, therefore, serves to highlight some common issues, to provide guidance for a priori time window and electrode selection, and to advocate for more rigorous methodology and more comprehensive reporting in future.

This systematic review, although extensive, is far from exhaustive. Picture-evoked N400 is not the only ERP measure, and many methodology decisions were not considered in this review – from statistical power, to study design and hypotheses, participant exclusion criteria, compliance of graphs with recommendations for appropriate visualization of ERP data, details of more complex statistical analyses, and others. These questions remain to be explored in future studies.

In addition to expanding the scope of the literature review, two additional questions naturally come to mind. The first question is—how much does the observed variability in pre-processing and analysis pipelines affect our knowledge about the N400? One way to answer this question is to implement Multiverse Analysis approach (Steegen et al., 2016) to examine to what extent the variability present in the N400 literature affects results of experiments (e.g., Author(s); Kappenman & Luck, 2010; Tanner et al., 2015). Regardless of the outcomes of such analyses, basing a priori decisions about the N400 window, locations or measurement reference on the existing literature, as suggested in this paper, would improve coherency and comparability between future reports.

The second question is—what we can do to improve reporting on the N400, and more broadly ERP, studies. For one, we hope that future researchers, especially the ones who are just diving into the field of ERP research, will find our account of the most frequently omitted items and examples of wordings that are insufficiently informative helpful. Secondly, given the amount of detail that is required for a thorough report on ERP data recording and pre-processing, it is challenging to fit everything in a typical journal article format, which is why researchers were often in position to choose which aspects they can describe in more details, and which they need to shorten as much as possible. While it is also important to strive to provide as accurate and as detailed report in a journal paper, the more recent availability of online repositories for supplementary materials helps overcome this challenge by providing additional space for all information that cannot fit within a given limit of characters available for the paper itself. Finally, several initiatives which call for action and propose a solution in the form of checklists and reporting templates have arisen in the past few years (Gau et al., 2021; Keil et al., 2014; Pernet et al., 2018). To advance this effort within ERP specifically, the item-level details arising from this systematic review have been adapted into a reporting template designed to make reporting easier and more accurate: Agreed Reporting Template for EEG Methodology - International Standard (ARTEM-IS) for ERP research (Styles et al., 2021). Given the number of details that needs to be provided for an ERP study to be fully reproducible, these initiatives provide promising tools for reducing omissions and ambiguous reports on methodological details.

**Table 1:** Papers evaluated in this report, in chronological order by year and alphabetical order within a year

No	Decade	Study
1	1980s	(Barrett et al., 1988)
2		(Barrett & Rugg, 1989)
3	1990s	(Barrett & Rugg, 1990)
4		(Friedman, 1990)
5		(Nigam et al., 1992)
6		(Stuss et al., 1992)
7		(Bobes et al., 1994)
8		(Holcomb & McPherson, 1994)
9		(Perez-Abalo et al., 1994)
10		(Pratarelli, 1994)
11		(Nielsen-Bohlman & Knight, 1995)
12		(Schweinberger et al., 1995)
13		(Yano, 1995)
14		(Debruille et al., 1996)
15		(Ganis et al., 1996)
16		(Pietrowsky et al., 1996)
17		(Simos & Molfese, 1997)
18		(Mecklinger, 1998)
19		(Münte et al., 1998)
20		(Grigor, 1999)
21		(Jordan & Thomas, 1999)
22		(McPherson & Holcomb, 1999)
23		(Olivares et al., 1999)
24	2000s	(Castle et al., 2000)
25		(Eimer, 2000)
26		(Kiefer, 2001)
27		(Bensafi et al., 2002)
28		(Federmeier & Kutas, 2002)
29		(Hamm et al., 2002)
30		(West & Holcomb, 2002)
31		(Ganis & Kutas, 2003)
32		(Jemel et al., 2003)
33		(Mnatsakanian & Tarkka, 2003)
34		(Olivares et al., 2003)
35		(Schendan & Kutas, 2003)
36		(Wang et al., 2003)
37		(Wicha, et al., 2003a, 2003b)
38		(Wicha, et al., 2003a, 2003b)
39		(Gunter & Bach, 2004)
40		(Mnatsakanian & Tarkka, 2004)
41		(Trenner et al., 2004)
42		(Wang et al., 2004)
43		(Yovel & Paller, 2004)
44		(Balconi & Pozzoli, 2005)
45		(Gierych et al., 2005)
46		(Supp et al., 2005)
47		(Eddy et al., 2006)
48		(Paz-Caballero et al., 2006)
49		(Cooper et al., 2007)
50		(Mao & Wang, 2007)
51		(Proverbio et al., 2007)
52		(Wu & Coulson, 2007)
53		(Boldini et al., 2008)
54		(Hirschfeld et al., 2008)
55		(Koester & Schiller, 2008)
56		(Lüdtke et al., 2008)
57		(Neumann & Schweinberger, 2008)
58		(Ortega et al., 2008)
59		(Steffensen et al., 2008)
60		(Zhang et al., 2008)
61		(Eddy & Holcomb, 2009)
62		(Harris et al., 2009)
63		(Kovic et al., 2009)
64		(Proverbio & Riva, 2009)
65		(Shibata et al., 2009)
66	2010s	(Eddy & Holcomb, 2010)
67		(Khateb et al., 2010)
68		(Liu et al., 2010)
69		(Lu et al., 2010)
70		(Mudrik et al., 2010)
71		(Olivares & Iglesias, 2010)
72		(Proverbio et al., 2010)
73		(Saavedra et al., 2010)
74		(Eddy & Holcomb, 2011)
75		(Herring et al., 2011)
76		(Huffmeijer et al., 2011)
77		(Kiefer et al., 2011)
78		(Kuipers & Thierry, 2011)
79		(Liao et al., 2011)
80		(Lin et al., 2011)
81		(Maillard et al., 2011)
82		(Wu & Coulson, 2011)
83		(Yum et al., 2011)
84		(Blackford et al., 2012)
85		(Bramão et al., 2012)
86		(Cansino et al., 2012)
87		(Cohn et al., 2012)
88		(Demiral et al., 2012)
89		(Hirschfeld et al., 2012)
90		(Kovalenko et al., 2012)
91		(Schendan & Ganis, 2012)
92		(Butler et al., 2013)
93		(Diéguez-Risco et al., 2013)
94		(Giglio et al., 2013)
95		(Olivares et al., 2013)
96		(Proverbio et al., 2013)
97		(Riby & Orme, 2013)
98		(Võ & Wolfe, 2013)
99		(Baetens et al., 2014)
100		(Balconi & Vitaloni, 2014)
101		(Boutonnet et al., 2014)
102		(Lensink et al., 2014)
103		(Li & Lu, 2014)
104		(Manfredi et al., 2014)
105		(Mudrik et al., 2014)
106		(Proverbio et al., 2014)
107		(Schleepen et al., 2014)
108		(Dominguez-Martinez et al., 2015)
109		(Dyck & Brodeur, 2015)
110		(Gao et al., 2015)
111		(Kaczer et al., 2015)
112		(Khushaba et al., 2015)
113		(Küper et al., 2015)
114		(Maffongelli et al., 2015)
115		(Ousterhout, 2015)
116		(Proverbio et al., 2015)
117		(Schendan & Ganis, 2015)
118		(Zani et al., 2015)
119		(Zhou et al., 2015)
120		(Hoogeveen et al., 2016)
121		(Niu et al., 2016)
122		(Rojas et al., 2016)
123		(Yinan Wang & Zhang, 2016)
124		(Gui et al., 2017)
125		(Kiefer et al., 2017)
126		(Mandikal Vasuki et al., 2017)
127		(Ortiz et al., 2017)
128		(Pergola et al., 2017)
129		(Savic et al., 2017)
130		(Wang et al., 2017)
131		(Bouten et al., 2018)
132		(Yi et al., 2018)

## Supplementary Information

Below is the link to the electronic supplementary material.

Supplementary file1 (DOCX 14 KB)

## Footnotes

1 We compared the N400 measurement and analysis montage of aging studies and studies including targets in other modalities, but there were no discrepancies from the overall results.

2 In some cases, there was more than one experiment in a paper. Furthermore, individual experiments could have uneven groups or an uneven number of trials per condition. In these situations, we chose the lowest number, because we were interested in how often publications deviated from the guidelines for good practice.

3 One point that the N400 researchers may want to pay attention to in the future is reporting on excluded participants. In a handful of cases, the sample description did not allow determining whether the sample size was given with or without excluded participants.

4 This difference remains after removing three outliers with more than 40 ANOVAs.

5 Seventy-four properties were extracted, but publication details, such as paper type (article vs. proceedings) were not included.
