# Supplementary material for: How to do Better N400 Studies: Reproducibility, Consistency and Adherence to Research Standards in the Existing Literature
Source: Neuropsychol Rev. 2021 Aug 9;32(3):577–600. doi: 10.1007/s11065-021-09513-4 (PMC9381463; doi:10.1007/s11065-021-09513-4)
Supplement: Supplementary file 1 — Supplementary file1 (DOCX 14 KB) [file 11065_2021_9513_MOESM1_ESM.docx]

Supplementary materials

Supplementary materials to this paper can be found on the project’s Open Science

Foundation page (https://osf.io/n426j/) and they include: a list of exact search strings used to search Web of Science and PubMed databases (Supplement 1); libraries with papers found by searching

these databases (Supplement 2a) and papers that were selected for analysis (Supplement 2b); the

Codebook with information on all variables used to analyse papers (Supplement 3); PRISMA

Checklist (Supplement 4); a spreadsheet with all variables and information on individual papers

(Supplement 5); files containing all analyses and graphs (Supplement 6: 6a, 6b, 6c, 6d); documents

with detailed results on all aspects of research methodology included here (Supplement 7: 7a, 7b,

7c, 7d).
